# Technological Progress in Generation of Induced Pluripotent Stem Cells for Clinical Applications

**DOI:** 10.1100/2012/417809

**Published:** 2012-03-12

**Authors:** Seung-Ick Oh, Chang Kyu Lee, Kyung Jin Cho, Kyung-Ok Lee, Ssang-Goo Cho, Sunghoi Hong

**Affiliations:** ^1^Department of Biomedical Science, College of Health Science, Korea University, Jeongneung-dong, Sungbuk-gu, Seoul 136-703, Republic of Korea; ^2^Division of Stem Cell Research NeoDIN Medical Institute, Yongdap-dong, Sungdong-gu, Seoul 133-847, Republic of Korea; ^3^Department of Animal Biotechnology and Animal Resources Research Center, Konkuk University, Seoul 143-701, Republic of Korea

## Abstract

Reprogramming of somatic cells into induced pluripotent stem cells (iPSCs) is achieved by viral-mediated transduction of defined transcription factors. Generation of iPSCs is of great medical interest as they have the potential to be a source of patient-specific cells. For the eventual goal of clinical application, it is necessary to overcome the limitations of low reprogramming efficiency and chromosomal abnormalities due to viral DNA integration. In this paper, we summarize the current state of reprogramming technology for generation of iPSCs and also discuss potential approaches to the development of safe iPSCs for personalized cell-based replacement therapy.

## 1. Introduction

Embryonic stem cells (ESCs), which are derived from the inner cell mass of blastocyst stage embryos, have the unique ability to self-renew indefinitely as well as the capability to differentiate into three germ lineages, which eventually give rise to the various cell types of the human body [1, 2]. Human embryonic stem cells can provide a potential source of cells for cell replacement therapy and/or drug discovery for the treatment of disastrous disorders, but there are limitations that must be overcome, such as immune rejection and ethical issues surrounding the use of human embryos as an ESC source, for hESCs to be used clinically [3].

Cell differentiation into specific cell types is considered to be unidirectional, as cell reprogramming has been rarely observed [4, 5]. However, nuclear transfer and cell fusion experiments have demonstrated that somatic cells could be reprogrammed into a pluripotent embryonic cell state through the epigenetic modifications [6, 7] while these technologies still require the use of embryos.

A major advance in the stem cell field was the conversion of somatic cells to an embryonic stem cell state, which was named as induced pluripotent stem cells (iPSCs), using defined transcription factors by Yamanaka and colleagues in 2006 and 2007. iPSCs can avoid immune rejection, since cells are derived from a patient's own cells, as well as any ethical issues regarding the use of human embryos. The characteristics of iPSCs are also very similar to those of pluripotent ESCs in many aspects, including cell morphology, expression of pluripotent markers, patterns of epigenetic changes, and capability to form embryoid bodies, teratoma, and viable chimeras [8]. However, there are still a number of problems related to current reprogramming methods. The use of viral vectors has led to the integration of multiple viruses into iPSC genomes, resulting in tumorigenesis due to genetic abnormalities in the cells. The reprogramming efficiency of human iPSCs from fibroblasts is very low, approximately less than 0.02% [9]. The use of Myc gene as a reprogramming factor and/or the reactivation of a silenced Myc gene might cause iPSCs to become cancer cells. The kinetics for reprogramming of human iPSCs are also very slow, taking more than 3 weeks approximately [10]. Both the low efficiency and slow kinetics of iPSC reprogramming may result in genetic alterations that affect the pluripotent and differentiation properties of iPSCs and ESCs. Addressing these concerns is already a top priority in this field.

Many groups have designed more efficient and safer reprogramming methods for iPSC generation than Yamanaka's protocol. In this paper, we summarize various reprogramming methods and also discuss the main approaches to achieving safe iPSC generation for regenerative medicine.

## 2. Reprogramming by Nuclear Transfer

Nuclear transfer constitutes a proof of principle that reversible genomic alterations are required for normal development. However, since there were no factors reported in previous reprogramming studies, there remains a question as to whether or not terminally differentiated cells can be reprogrammed into a totipotent state. The successful generation of genetically identical mouse clones by somatic cell nuclear transfer (SCNT) technology from various mature cell types [11–13] has demonstrated that terminally differentiated cells have the nucleus potential to support development. Importantly, the reprogramming of somatic donor cells using SCNT also has revealed that unfertilized eggs contain pluripotent genes [14]. Cloning from terminally differentiated donor cells is inefficient, with successes coming only when cloned ESCs are used. Binucleate hybrid cells produced by cell fusion of embryonic cells with somatic cells have been used to demonstrate the epigenetic reprogramming of somatic cells to a pluripotent state. Mouse and human hybrid cells produced by fusion between various somatic cells and embryonic carcinoma cells [15], embryonic germ cells [16] or ESCs [7, 16, 17] share the same phenotype and gene expression pattern as parental embryonic cells, which indicates that ESCs express dominant pluripotent factors for reprogramming of somatic cells. Therefore, nuclear reprogramming studies using SCNT and cell fusion have demonstrated that transcriptional factors are essential for the reprogramming of terminally differentiated cells. 

## 3. Reprogramming by Defined Transcription Factors

In 2006, Takahashi and Yamanaka achieved a breakthrough in the reprogramming of somatic cells to a pluripotent ESC-like state through the transduction of retroviral vectors containing 24 candidate genes into mouse fibroblasts. The pool of genes was finally narrowed down to four transcription factors, Oct3/4, Sox2, c-Myc, and Klf4, which were transduced into mouse fibroblasts containing a fusion cassette of the **β**-galactosidase and neomycin resistance genes downstream of the *Fbx15 *gene promoter. The transcription factors were already known to contribute to the self-renewal of pluripotent ESCs. The *Oct-4*(*Pou5f1*) gene, named as Octamer (ATGCA/TAAT)-binding protein-*4*, encodes a transcription factor that belongs to the class of POU factors (known as *Pit1*, *Oct1*, *Oct2*, and *Unc86*), which have a bipartite DNA-binding domain [40]. Oct4 is known to be required for the formation of the inner cell mass in early embryos and for the maintenance of the pluripotency of ESCs [41–43]. Oct4 can also form a homodimer by itself as well as a heterodimer with Sox2, and its cooperative binding with Sox2 promotes the transcriptional regulation of various target genes such as Nanog [44]. Interestingly, although Scholar's group demonstrated that Oct4 alone can reprogram mouse and human neural stem cells to iPSCs [45–47], a two-factor combination of Oct4 and Sox2 or a three-factor combination of Oct4, Sox2, and Klf4 is still required for iPSC generation in most somatic cell types. The *Sox2 *gene, designated as *S*RY- (*S*ex-determining Region Y-) b*ox2*, encodes a transcription factor belonging to the Sox family of proteins, which bind to DNA through their 79-amino-acid HMG domain [48]. The *Klf4* gene, named as Krüppel-like zinc-finger protein *4*, encodes a transcription factor and is known to be required for establishment of left-right asymmetry in early embryos. Klf4 can also directly bind to the Oct4-Sox2 heterodimer in mouse ESCs, and tetrameric complexes containing the DNA element of a target gene are required for somatic cell reprogramming [49]. The *c-Myc *gene, named as cellular homolog of retroviral v-*myc* oncogene, encodes a transcription factor containing a basic helix-loop-helix/leucine zipper domain. c-Myc binds to DNA through its bHLH motif and heterodimerizes with other interacting proteins through its leucine zipper motif. c-Myc is involved in the maintenance of pluripotent ESCs through signaling [50] and promotes cell proliferation by inducing global histone acetylation by histone acetyltransferases (HATs). Other groups have reported that only the three transcription factors Oct4, Sox2, and Klf4 can successfully generate iPSCs from mouse and human fibroblasts [51], indicating that c-Myc is not decisively essential for direct reprogramming of somatic cells into iPSCs. iPSCs derived from these three factors do not develop into cancer cells, which is counter to a report that cancer developed in 20% of chimeric mice [52]. The use of subtypes of transcription factors was used successfully to generate iPSCs from somatic cells, although reprogramming efficiency was affected; Sox2 can be replaced by Sox1 and Sox3, c-Myc can be replaced by L-Myc and N-Myc, and Klf4 can be replaced by Klf2 [51, 53]. Thomson's group used a different set of transcription factors containing additional two reprogramming factors such as Nanog and Lin28 to reprogram human fibroblasts [23]. The *Nanog *gene, named after Tir Na Nog, encodes a transcription factor containing a conserved NK2-family homeodomain motif. Nanog is expressed in pluripotent stem cells and is involved in cell proliferation and maintaining self-renewal of ESCs [54]. Nanog can also assemble into homodimers with itself through a specialized tryptophan-rich C-terminal domain for the cooperative regulation of target genes [55, 56]. *Lin28* (*Lin*-*28* homolog A) encodes a cytoplasmic mRNA-binding protein that can drive specific mRNAs to translational machinery for the enhancement of protein synthesis [57]. In order to reprogram mature cells into iPSCs, many modified reprogramming protocols using different combinations, different subtypes, and different sets of transcription factors have been used to date. However, we need to realize that some of these factors are oncogenes, which may cause tumor formation in the form of oncogene-derived iPSCs. 

## 4. Reprogramming Technologies

**Figure 1:**
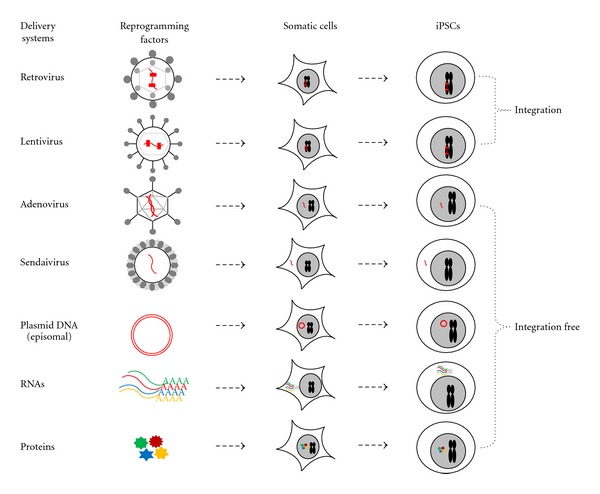
strategies for generation of somatic cells into induced pluripotent stem cells (iPSCs) using different gene delivery systems.

The choice of a gene delivery system is a key aspect for iPSC generation. Many researchers in this field still use viral or nonviral methods to reprogram mature cells (Figure 1), and some groups have tried to use nongenetic materials for the generation of efficient and safe iPSCs (Table 1). 

### 4.1. Integration Viral Vector Systems

For the initial generation of iPSCs, retroviral vectors as a powerful gene delivery system have been used to introduce the four transcription factors into fibroblasts. Retroviral vectors can be efficiently transduced into target cells and randomly integrated into the host genome of only dividing cells. Although retroviral vectors have higher efficiency of transduction than that of other viral vectors without any severe effects on cell viability, the expression of integrated genes could become silenced during the epigenetic processes of gene regulation, which provides an advantage in that the temporal expression of exogenous factors may be required for the generation of iPSCs. As another gene delivery system for the generation of iPSCs, lentiviral vectors have been used. Lentiviral vectors as a subclass of retroviruses can also be integrated into the host genome of both dividing and nondividing cells, which means that lentiviral vectors can be applied to a wide variety of cell types. In addition, advanced inducible-lentiviral vector systems using doxycycline as an inducer have been used for the specific control of the expression of the four transcription factors and for high efficiency of fully reprogrammed iPSCs during induction [27, 58–60]. Although efficient gene delivery systems using retroviral and/or lentiviral vectors have been used to introduce reprogramming factors into somatic cells, these viral vector systems remain controversial due to multiple copies of proviral genomic integration, which may cause both the reactivation of silenced exogenous oncogenes such as Klf4 and c-Myc and the alteration of genomic construction, thereby increasing the risk for malignant cancer transformation [60]. Efforts for the practical use of iPSCs in clinical applications have led to the technical development of nonintegration viral vectors or DNA approaches.

### 4.2. Integration-Free Viral Vectors and DNA Systems

#### 4.2.1. Viral Vectors

Using an adenoviral vector as a dsDNA virus, which is a nonintegration vector that remains in epichromosomal form in cells, virus-free iPSCs have been successfully generated, although with low efficiency [27, 59, 60]. In addition, since the adenoviral vector system gives rise to only transient expression, it may require the repeated delivery of reprogramming factors during induction of iPSCs. However, the success of nonintegrating iPSC generation suggests that the integration of transcription factors into the genome is not required for the reprogramming of somatic cells, providing the potential to develop nonviral delivery technologies that could be used to generate safe iPSCs. As another viral vector system without genomic integration, Sendaiviral (Sendaivirus) vector, which is an RNA virus that replicates its genome exclusively in the cytoplasm, efficiently generated iPSCs from human somatic cells [28]. Nonetheless, iPSC research is moving toward the development of new technologies without genetic modification. 

#### 4.2.2. Plasmid DNA and Transfection

A nonviral system is transfection technology that uses complexes of plasmid DNA carrying reprogramming factors and lipid or cationic polymers that are then introduced into the cells to be reprogrammed. Plasmid DNA is not usually integrated into the host genome and exhibits gene expression after 2-3 days. Okita et al. reported the first successful generation of iPSCs from mouse and human fibroblasts by repeated transfection of two expression plasmids in a nonviral vector system, one expressing Oct4, Sox2, and Klf4 and the other expressing c-Myc [61]. Most iPSC lines generated by this method are free from DNA integration into the host genome, even though plasmid DNA integration in some lines has been detected at rates as low as approximately 5.5%. The advantage of this method is the generation of iPSCs without plasmid DNA integration at a rate as high as 60%, indicating reproducibility of the transfection technique. However, the efficiency of iPSC generation is substantially lower than that of viral systems using retroviral or lentiviral vectors, which may be due to lower transfection efficiency or transgene expression levels [29]. Another group successfully generated iPSCs without any plasmid DNA integration from mouse embryonic fibroblasts using a single plasmid DNA containing all four factors through nucleofection transfection technique, which is based on the electroporation method generated by a Nucleofector device [62]. Unlike regular plasmid vectors, oriP/EBNA1 episomal plasmid vectors derived from Epstein-Barr virus can be transfected without the need for viral packaging, although they can be replicated only once per cell cycle [63, 64]. This vector can be established as a stable episome in transfected cells through drug selection, but it is not capable of integration into the host cell genome [31, 65, 66]. If drug selection is absent, the vectors are gradually lost at rate of a 5% during cell division due to defects in plasmid synthesis, which allows cells lacking plasmids to be easily isolated [67]. Yu et al. successfully generated the first human iPSCs using an oriP/EBNA1 episomal plasmid containing reprogramming factors, but reprogramming efficiency was extremely low (3 to 6 colonies per 10^6^ input of cells) [31]. Recently, Jia et al. reported the successful generation of iPSCs from adult human adipose-derived stem cells (ADSCs) by nucleofection of minicircle vector DNA. The minicircle vectors are supercoiled DNA molecules devoid of any bacterial plasmid DNA backbone. Their smaller molecular size allows for more efficient transfections and offers sustained expression over a period of weeks as compared to regular plasmid vectors, which only work for a few days [35]. However, the overall reprogramming efficiency is very low (approximately 0.005%). Very recently, our group successfully generated DNA-free iPSCs magnetically by introducing complexes of regular plasmid DNA containing each factor and nanoparticles into mouse fibroblasts, although some iPSC lines were detected with genomic integration of the plasmid DNA and the reprogramming efficiency was relatively low [30]. 

#### 4.2.3. Excision of Integrated Transgenes

There are two systems that use either Cre/*loxP* recombination or *piggyBac* transposition for the removal of exogenous reprogramming factors from genomic integration sites in iPSCs. In the Cre/loxP recombination system, a loxP site is inserted into the 3′ long-term repeat (LTR) of self-inactivating (SIN) lentiviral vectors containing reprogramming factors under the control of a doxycycline- (Dox-) inducible minimal cytomegalovirus (CMV) promoter [68]. The loxP is duplicated into the 5′LTR during proviral replication, resulting in host genomic integration with a transgene flanked by two loxP sites. The lentiviral vector system has been used to generate iPSCs with multiple copies of genomic integration, after which the iPSCs are transiently transfected with an expression vector encoding Cre recombinase and the puromycin resistance gene by electroporation, thus enabling the excision of all reprogramming factors [68–70]. Although the resulting factor-free iPSCs show a similar gene expression profile as that of ESCs rather than that of the preexisting iPSCs, the Cre-mediated excision protocol leaves behind a loxP and vector DNA fragment in the iPSCs that can result in genomic instability and genome rearrangements. To address these multiple genomic integrations, Shao et al. developed a single plasmid vector system with a 2A-peptide-linked reprogramming cassette originating from the foot-and-mouth disease virus of the Picornaviridae family to generate virus-free, factor-removable iPSCs [33, 71]. The multiprotein expression system was shown to minimize genome modification in iPSCs and increase reprogramming efficiency, but it still displayed the same problems as the Cre/loxP recombination system. As an alternative strategy to excise remaining exogenous DNA, Yusa et al. recently used the *piggyBac *(PB) transposon/transposase system, which is capable of removing itself precisely from cells, to successfully generate iPSCs bearing a single integration site from somatic cells [34]. The PB plasmid vector was constructed with 2A peptide-linked reprogramming factors under the control of the tetO tetracycline/doxycline inducible promoter, which was inserted between PB 5′ and 3′ terminal repeats. Moreover, the original integration sites were subsequently excised from the iPSCs at a rate of higher than 90% by transient expression of PB transposase. O'Malley et al. also used PB-based vectors constructed with *puΔtk *cassette as a negative selection marker and 2A peptides-linked reprogramming factors under the control of the CAG promoter to efficiently generate integration-free iPSCs from mouse fibroblasts after transient PB treatment [72]. However, these excision approaches are complex and time-consuming since they require the identification of iPSCs with minimal-copy insertions, mapping of integration sites, excision of the reprogramming cassette, and validation of factor-free clones [73].

## 5. Approaches to Enhancement of Reprogramming Efficiency

Despite the successful generation of iPSCs using reprogramming factors, the slow and inefficient nature of the reprogramming process initially limited the generation of iPSCs as well as their potential clinical application. However, a number of groups recently reported studies on the improvement of reprogramming efficiency using different reprogramming factors either alone or in combination with chemicals. Park et al. increased reprogramming efficiency approximately 3-fold using SV40 large antigen (SV40LT) and human telomerase reverse transcriptase (hTERT) as additional factors in combination with Oct, Sox2, Klf4, and c-Myc (OSKM) [74, 75]. Maekawa et al. increased reprogramming efficiency about 70-fold using a different combination of transcription factors such as SV40 TL, Oct4, Sox2, Nanog, and Lin28 [76]. Very recently, Zhao et al. observed that the Gli-like transcription factor Glis1 (Glis family zinc finger 1) markedly enhanced the generation of iPSCs from mouse and human fibroblasts transduced with three factors (OSK) [77]. Strikingly, human fibroblasts transduced with lentiviral vectors containing OSKM, p53 siRNA, and *UTF1* previously showed more than 100-fold enhancement of reprogramming efficiency as compared to those transduced only with OSKM. In addition, when p53 siRNA and *UTF1* were added to a combination of three factors (OSK), the reprogramming efficiency increased 100-fold compared to treatment with only OSK [78, 79]. These data suggest that *UTF1*, which is known to be a target of the Oct4-Sox2 heterodimer [80], can activate other important downstream genes for reprogramming [78, 79]. As an explanation, *p53* loss may enhance reprogramming efficiency through stimulation of cell cycle progression by inhibiting both cell death and senescence, but there is a risk that the iPSCs are abnormal since p53 is known to play a crucial role in the maintenance of genomic integrity. Recently, these suggestions have been demonstrated experimentally and the roles of p53 were actively described during the reprogramming process [81–87]. However, Mikkelsen et al., who used pre-B cells derived from NGFP1 iPSCs, which had been transduced with p53 siRNA, reported that the key parameter of reprogramming efficiency is the number of cell divisions [88]. A combined approach using transient p53 suppression with reprograming factors in an integration-free delivery system [61] or with chemicals known to modulate genome-wide chromatin structure and gene activities [89–91] was previously shown to increase reprogramming efficiency for therapeutic use. 

Besides reprogramming factors and chemicals as epigenetic modifiers, the microRNAs (miRNAs) are known to play an important role in the reprogramming process and efficiency [92]. Recently several groups efficiently generated iPSCs from mouse and human fibroblasts using miRNAs [93, 94]. In addition, Zhou et al. showed that the miR302/367 cluster efficiently can reprogram mouse and human fibroblasts into an iPSC state without using any reprogramming factors [95]. However, all of the iPSCs made using either miRNAs alone or in combination with the four factors were integrated by retroviral or lentiviral vectors. 

## 6. Approaches for Safe and Efficient Reprogramming

All the methods that have been used to date still involve the use of genetic transcription factors, which could cause potential risks of tumorigenesis. However, various approaches to avoid the introduction of exogenous genetic factors to target cells have been also developed as discussed above, and recent studies have provided attractive methods using reprogramming proteins, mRNAs, and chemicals to address safety concerns.

Two groups demonstrated that purified recombinant reprogramming factor proteins fused with a polyarginine cell-penetrating peptide (CPP) can successfully generate iPSCs from mouse and human fibroblasts [45–47, 96]. Ever since trans-activating transcriptional activator (TAT) peptide was first isolated from human immunodeficiency virus 1 (HIV-1) in 1988 [97], CPP technology has been used to promote the cellular uptake of molecular cargos, such as small chemical molecules, DNAs, or proteins. Frankel and Pabo transduced reprogramming proteins into target cells for four cycles overnight in combination with valproic acid (VPA) and an HDAC inhibitor, followed by 36 additional hours of culturing, after which the treated cells were transferred into irradiated feeder cells and kept in ESC media until three iPSC colonies formed between days 30 to 35 [96]. Kim et al. transduced reprograming proteins for six cycles over 16 hours, followed by 6 days of culturing, and successfully established two iPSC lines out of five colonies isolated on day 56 [45–47]. Although the protein-based reprogramming approach successfully generated iPSCs that were not integrated by any genetic factors, the reprogramming process was very slow and efficiency was very low, causing some changes in genomic integrity. In addition, the requirement for the multiple transduction of reprogramming factor proteins hampered the reprogramming process.

Yisraeli and Melton generated iPSCs using mRNAs of the reprogramming factors that were synthesized by *in vitro* transcription (IVT) of the PCR amplicons and then additionally modified with a cap analog and poly-A tail to promote the initial binding of ribosomes and mRNA stability in the cytoplasm [38, 98]. Although this synthetic RNA-based approach safely and efficiently induced iPSCs, it was technically complex with multiple transfections required. Another alternative approach to safely improve the reprogramming process for the generation of iPSCs is to use a cocktail of small molecules that are linked with epigenetic modifiers and major signaling pathways. Inhibitors of histone deacetylases (HDACs), histone demethylases (HDMs), and histone methyltransferases (HMTs), which regulate chromatin remodeling, have been identified as small molecules for reprogramming somatic cells into iPSCs. HDAC inhibitors such as VPA, trichostatin A (TSA), and suberoylanilide hydroxamic acid (SAHA) significantly were shown to improve reprogramming efficiency. In particular, VPA treatment greatly improved reprogramming efficiency by more than 100-fold in four factor-infected MEFs hemizygous for the Oct4-GFP transgene as a reporter [99]. The Wnt signaling pathway is involved in promoting self-renewal of ESCs through inhibition of GSK-3*β* and subsequent nuclear accumulation of *β*-catenin [100]. Interestingly, when Dox-inducible OSK-MEFs were previously treated with Wnt3a-conditioned medium, reprogramming efficiency increased by as much as 20-fold [101]. Lin et al. reported that inhibition of GSK-3*β* using CHIR99021 significantly improved the reprogramming efficiency of MEFs transduced with three factors (OSK) [102]. In addition, Takahashi reported that combined inhibition of the TGF*β* and MEK-ERK pathways using SB-431542 and PD-0325901, respectively, not only improved the efficiency of the reprogramming process but also accelerated kinetics. When a cocktail of SB-431542 and PD-0325901 including thiazovivin, which improves the survival of human ES cells upon trypsinization (unpublished data by Ding's group), was treated to human fibroblasts transduced with the four factors, the efficiency of the reprogramming process was dramatically improved more than 200-fold [39].

However, there still remain safety concerns in terms of more subtle and harmful endogenous genetic and epigenetic alterations that may occur during reprogramming of iPSCs, since cell growth pathways could be activated and tumor suppressor pathways could be also inhibited after using a small molecule or a cocktail of small molecules.

## 7. Conclusion

The reprogramming of somatic cells into pluripotent cells was originally achieved by transduction of retroviral vectors containing four transcription factors into mouse and human fibroblasts [9, 21, 60, 102]. Although recent studies have shown significant technical progress in improving reprogramming, simple and efficient reprogramming approaches are still strongly required. In addition, the development of technology to generate iPSCs for clinical applications should address these safety concerns. 

The iPSC field should focus more on both the development of more advanced reprogramming technologies that employ a nonintegrating vector carrying a minimal set of reprogramming factors and the identification of new small molecules that modulate the reprogramming process. The combinatorial action of epigenetic or signaling modifiers with nonintegrating delivery systems containing reprogramming factors could be a powerful approach to generate more efficient and safer iPSCs. However, it still remains a challenge to reprogram somatic cells only by treatment with small molecules or manipulation of cell culture conditions while overcoming potential risks. 

## Figures and Tables

**Table 1:** Methods of efficient and safe iPSCs generation for clinical applications.

Methods	Advantage	Disadvantage	Species	Efficiency (%)	Safety	References
Retroviral vectors	Dividing cell infection, moderate efficiency	Multiple integration, incomplete silencing, tumorigenicity possible	P, R, Rh, M, H	0.01*∼*0.5	No good	[18–21]
Lentiviral vectors	Dividing or nondividing cell infection, moderate efficiency	Multiple integration, incomplete silencing, tumorigenicity possible	M, H	0.1*∼*1	No good	[22, 23]
Induced lentiviral vectors	Tight transcriptional regulation, dividing or nondividing cell infection, moderate efficiency	Multiple integration, transactivator needed, tumorigenicity possible	P, M, H	0.1*∼*1	No good	[24–26]
Adenoviral vectors	Nonintegration	Integrated vector-fragment possible, low efficiency	M, H	*∼*0.001	Good	[27]
Sendaiviral vectors	Transgene decreased during cell division, nonintegration, easy to remove Sendaivirus	Integrated vector-fragment possible	H	0.001*∼*1	Good	[28]
Plasmids	Simple transfection	Occasional integration, low efficiency	M, H	*∼*0.001	Good	[29]
Plasmids + Nanoparticles	Rapid and simple transfection	Occasional integration, low efficiency	M	0.001*∼*0.003	Good	[30]
oriP/EBNA-1 episomal vectors	Nonintegrating vector, long-term persistent transcription	Extremely low efficiency	H	*∼*0.0003	Good	[31]
Cre/loxP recombination systems	Integration but excisable, dividing or nondividing cell infection	Inefficient loxP site excision, screening needed, tumorigenicity possible	H	0.1*∼*1	No good	[32]
Piggyback transposon/transposase system	Precise excision possible, moderate efficiency	Screening needed,	M, H	*∼*0.1	Good	[33, 34]
Minicircle DNA episomal vectors	Improved efficiency, nonintegration	Low efficiency	H	*∼*0.005	Good	[35]
Proteins	DNA-free	Extremely low efficiency, long-term treatment required, genetic abnormality possible	M, H	*∼*0.001	Very good	[36, 37]
RNAs	DNA-free, High efficiency	Multiple transfection	H	*∼*1	Very good	[38]
Factors + Small molecules	High efficiency	Long-term treatment required, abnormal signaling pathway possible, virus used	M, H	*∼*2.05	No good	[39]

Pig (P); rat (R); rhesus monkey (Rh); mouse (M); human (H).
